# Nurse-based counselling on thirst in patients with advanced chronic heart failure: Study protocol for a pilot before–after study and process evaluation

**DOI:** 10.1007/s00063-023-01091-y

**Published:** 2023-12-14

**Authors:** Franziska Wefer, Ralph Möhler, Martin N. Dichter, Andrea Mühring, Jan Gummert, Sascha Köpke

**Affiliations:** 1 Care Development, Care Directorate, Heart and Diabetes Center NRW, University Hospital of the Ruhr University Bochum, Bad Oeynhausen, Germany Georgstr.11, 32545; 2 Institute of Nursing Science, Medical Faculty and University Hospital Cologne, University of Cologne, Gleueler Straße 176–178, 50935 Cologne, Germany; 3 Institute for Health Services Research and Health Economics, Centre for Health and Society, Medical Faculty and University Hospital Düsseldorf, Heinrich-Heine University, Düsseldorf, Germany; 4 Transplantation unit, Heart and Diabetes Center NRW, University Hospital of the Ruhr University Bochum, Bad Oeynhausen, Germany; 5 Clinic for Thoracic and Cardiovascular Surgery, Heart and Diabetes Center NRW, University Hospital of the Ruhr University Bochum, Bad Oeynhausen, Germany

**Keywords:** Complex interventions, Evidence-based, Nursing, Study protocol, Symptom, Komplexe Interventionen, Evidenzbasiert, Pflege, Studienprotokoll, Symptom

## Abstract

**Background:**

Many patients with chronic heart failure (CHF) are critically ill and experience increased thirst. Study aims are to develop and evaluate a nurse-based counselling intervention to promote self-care competencies related to thirst in hospitalised patients with advanced CHF eligible or listed for heart transplantation.

**Methods:**

A mixed-methods approach will be adapted with three study phases: (1) development of the nurse-based counselling intervention, (2) feasibility testing and training of nurses, and (3) implementation of the intervention and, evaluation of initial effects and process measures. In phase (1), interviews with hospitalised patients with advanced CHF listed for heart transplantation (*n* = 10), focus groups (*n* = 2) and a Germany-wide survey with nurses will be performed. In phase (2), experts experienced with caring for patients with advanced CHF and patients with advanced CHF will be consulted for content validation and pretest of the counselling intervention. The training concept for nurses will be evaluated using questionnaires. In phase (3), a pilot before–after study will be conducted (*n* = 60). Primary patient-related outcome for the pilot study is thirst intensity using a numeric rating scale. Furthermore, a process evaluation (interviews with patients [*n* = 10], survey with nurses and physicians) will be performed. Quantitative data will be analysed descriptively, and qualitative data will be analysed using content analysis. Mean values of thirst intensity of the individual measurement points will be evaluated as interrupted time-series analysis using regression analyses.

**Conclusion:**

The development and implementation of a counselling intervention is influenced by various factors. Therefore, it is important to consider all factors throughout the process from development to evaluation.

**Supplementary Information:**

The online version of this article (10.1007/s00063-023-01091-y) includes the SPIRIT 2013 Checklist.

## Abstract (de)

**Hintergrund:**

Viele Patienten mit chronischer Herzinsuffizienz (CHI) sind kritisch krank und leiden unter erhöhtem Durst. Ziel der Studie ist die Entwicklung und Evaluation einer pflegebasierten Beratungsintervention zur Förderung der Selbstpflegekompetenz bezogen auf Durst bei stationären Patienten mit fortgeschrittener CHI, die sich im Screening oder auf der Listung für eine Herztransplantation befinden.

**Methoden:**

Es wird ein Mixed-Methods-Ansatz mit 3 Studienphasen angewandt: (1) Entwicklung der pflegebasierten Beratungsintervention, (2) Machbarkeitsprüfung und Schulung von Pflegefachpersonen sowie (3) Implementierung der Intervention und Evaluierung erster Effekte und Prozessevaluation. In Phase (1) werden Interviews mit stationären Patienten mit fortgeschrittener CHI, die für eine Herztransplantation gelistet sind (*n* = 10), Fokusgruppen (*n* = 2) und eine deutschlandweite Umfrage mit Pflegefachpersonen durchgeführt. In Phase (2) werden Experten mit Erfahrung in der Versorgung von Patienten mit CHI und Patienten mit fortgeschrittener CHI zur inhaltlichen Validierung und zum Pretest der Beratungsintervention hinzugezogen. Das Schulungskonzept für die Pflegefachpersonen wird anhand von Fragebögen evaluiert. In Phase (3) wird eine Vorher-nachher-Pilotstudie durchgeführt (*n* = 60). Primärer patientenbezogener Outcome der Pilotstudie ist die Durstintensität anhand einer numerischen Ratingskala. Darüber hinaus wird eine Prozessevaluation (Interviews mit Patienten [*n* = 10], Befragung von Pflegepersonal und Ärzten) durchgeführt. Die quantitativen Daten werden deskriptiv, die qualitativen Daten inhaltsanalytisch ausgewertet. Die Mittelwerte der Durstintensität der einzelnen Messzeitpunkte werden als unterbrochene Zeitreihenanalyse mittels Regressionsanalysen ausgewertet.

**Schlussfolgerung:**

Die Entwicklung und Umsetzung einer Beratungsintervention können von verschiedenen Faktoren beeinflusst werden. Daher ist es wichtig, alle Faktoren während des gesamten Prozesses von der Entwicklung bis zur Evaluation zu berücksichtigen.

## Background

Patients with advanced chronic heart failure (CHF) are critically ill and often experience increased thirst due to their disease and therapeutic regiment [1]. Severe thirst can lead to distress and influence patients’ quality of life [15, 26]. More than 70% of patients with CHF may experience moderate to severe thirst distress [8, 26, 28]. Factors associated with thirst intensity are for example the severity of heart failure, fluid restriction and high doses of diuretics [25]. Adherence to fluid restriction is particularly relevant for those with advanced CHF and severe symptom burden [13].

A heart transplantation is one of the last therapeutic interventions for patients with advanced CHF. The waiting period is often associated with a long hospital stay, high emotional burden and strict adherence to dietary and treatment-related behaviours [5, 20, 21]. An increased feeling of thirst can influence the adherence to therapy-related interventions [24] and additionally increase patients’ stress during this time. Patients with CHF often lack options for alternative interventions to reduce thirst other than drinking fluids. In a study by van der Wal et al. [25] with 216 participants, 56% reported to drink more when they are thirsty, while 12% had no strategies to reduce thirst. Nine participants (4%) indicated sucking ice cubes as an intervention strategy and three participants preferred chewing gum [25]. A survey with health professionals (*n* = 42) in Australia found that the most recommended intervention was ice cubes (95%) [2].

Currently, there is little evidence about intervention strategies to reduce thirst in patients with CHF. A pilot study (*n* = 71) testing gum chewing as an intervention showed a significant reduction in thirst after 4 and 14 days [3]. Hudiyawati and Suswardany [10] analysed the effect of frozen strawberries compared with ice cubes (*n* = 34). Thirst intensity (visual analog scale [VAS], 0–10) decreased in both groups (*p* = 0.001). However, these studies present only limited evidence for a small number of interventions to alleviate thirst and do not address the individual needs and preferences of patients. Chewing gum as an example is not suitable for patients with swallowing disorders, while ice cubes can be painful for patients with sensitive teeth. Chen et al. [7] published a scoping review in which further single interventions, e.g. lemon water spray, ginseng plum decoction or black plum soup were mentioned. Studies were conducted in China and are not accessible.

Evidence-based counselling by nurses to promote self-care skills related to thirst in hospitalised critically ill patients with advanced CHF eligible or listed for a heart transplantation may support patients in obtaining information and selecting appropriate interventions to relief thirst. Self-care is a decision-making process that is influenced by actions, aiming to maintain physiological stability and facilitate symptom awareness and management [16]. It is also known that the onset of symptoms can negatively influence self-care behaviour [17]. For example, being stressed of feeling thirsty can have an impact on adherence to prescribed fluid restriction [24].

The development and evaluation of a nurse-based counselling intervention has not yet been investigated, neither nationally nor internationally.

## Aims and research questions

Aims of the study are (1) to develop a nurse-based counselling intervention to promote self-care competencies related to thirst in hospitalised patients with advanced CHF eligible or listed for heart transplantation, (2) to test the feasibility and (3) the initial effects of the intervention.

Research questions are

- How do hospitalised patients with advanced CHF experience their thirst? What are patients’ needs and requirements for information and what is their experience of health professionals providing information and advice on thirst as a symptom in the context of their disease? Which issues do they consider important in relation to thirst?
- Which information about thirst is provided by nurses on transplantation wards to patients with advanced CHF? Which issues do nurses consider important in relation to thirst?
- What is the feasibility of a counselling intervention to promote self-care competencies on thirst in hospitalised patients with advanced CHF failure eligible or listed for a heart transplantation?
- What are the initial effects of the counselling intervention and which factors influence its implementation?

## Methods

Data are reported following the recommendations for reporting study protocols of clinical intervention studies (SPIRIT) [6].

**Fig. 1:**
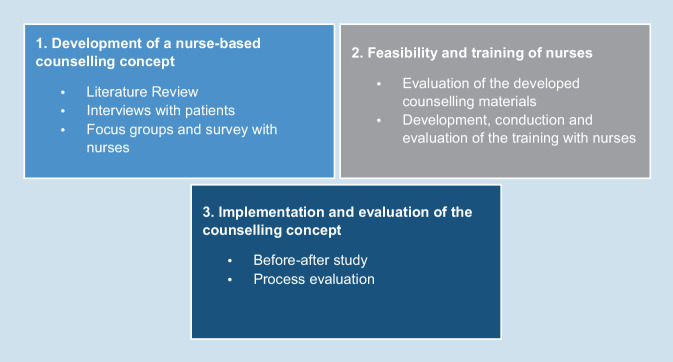
Study phases and methods

This study is based on the recommendations of the Medical Research Council (MRC) for the development and evaluation of complex interventions [19]. As recommended the research questions will be answered using a mixed methods approach. The project is divided into three study phases: (1) development of a nurse-based counselling intervention, (2) testing of feasibility and training of nurses and (3) implementation and evaluation of initial effects (Fig. 1).

**Fig. 2:**
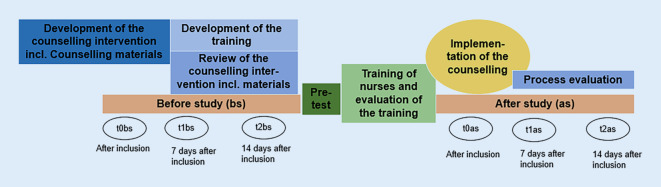
Study process including all study phases and measurement time points (t0-t2)

The study phases are not to be understood as strictly sequential phases as they can partly run in parallel. Figure 2 shows the overall study outline over time. In phase 1 (development of the counselling intervention), a systematic literature review (not reported here), 10 interviews with hospitalised patients with advanced CHF listed for heart transplantation, two focus groups with nurses and a Germany-wide survey with nurses will be performed. In phase 2 (feasibility of the counselling intervention), six to eight experts experienced in the care of patients with chronic heart failure (e.g. working group for Nursing Research in Cardiology/Cardiac Surgery, federal working group of nursing experts in heart failure in Germany) will be consulted for content validation. Subsequently, two guided interviews will be conducted with patients, who had a heart transplantation and experienced thirst during their disease. Goals of the interviews are to assess the comprehensibility, completeness and applicability of the counselling intervention. In addition, feasibility of the counselling intervention will be tested by two trained nurses on the wards of the included hospitals with four patients with advanced CHF waiting for a heart transplantation. To assess the feasibility and applicability of the intervention, a qualitative survey of the nurses and patients will be performed after the pretest of the counselling intervention. A training concept for nurses will be carried out and evaluated by means of a questionnaire.

This protocol focusses on the third phase, where the intervention will be implemented in practice and the initial effects of the new counselling intervention will be tested in a pilot before–after study. In addition, a process evaluation will be conducted. Data collection will be performed in two university hospitals.

## Pilot before–after study

The study will be designed as a pilot before–after study (interrupted time series analysis). Measurements will be conducted at three time points each before (before study [bs]) the training of the nursing staff (t0bs, t1bs, t2bs) and after (after study [as]) the implementation of the counselling intervention (t0as, t1as, t2as; Fig. 2).

### Sample and recruitment

Hospitalised patients with advanced CHF who are eligible or listed for heart transplantation will be included. Based on the study by Allida et al. [3], the case number estimate was calculated using a thirst intensity (NRS) of 4.2 ± 2 (baseline data). In order to detect a mean difference of one point with a standard deviation of 2 in thirst intensity (two-sided tests, significance level = 0.05 and power = 0.80), a sample of 67 patients per group is needed in a future randomised controlled trial. In order to determine feasibility and gather information for the future main effectiveness trial, we will first conduct a pilot study with a total of 60 patients (30 per group; two-sided tests, significance level = 0.30 [sic!] and power = 0.80).

Patients will be recruited consecutively. All patients treated at the heart failure units of the two participating university hospitals during the period of data collection who meet all inclusion criteria will be included in this study. Measurement will be conducted at two time points: before implementation of the counselling intervention (group I) and after implementation (group II).

### Inclusion and exclusion criteria

Patients with the following criteria will be included: advanced CHF, age ≥ 18 years, eligible or listed for heart transplantation, hospital stay (expected > 14 days), understanding and speaking the German language and available written informed consent.

Patients will be excluded if they are disorientated, mechanically ventilated, have an implemented (left) ventricular assist device (L)-VAD or are in the palliative phase. For the “after group”, patients who were already recruited for the “before group” will also be excluded.

### Outcome parameters

Primary outcome is thirst intensity, which is measured using a numerical rating scale (NRS; 0 = no thirst, 10 = strongest thirst).

Secondary outcomes are

- Thirst distress using the Thirst Distress Scale for patients with heart failure (TDS-HF) [29],
- Thirst frequency: At what time of day was the feeling of thirst strongest? (morning, afternoon, evening, night),
- Dry mouth with the short form of the xerostomia inventory (XI) [23],
- Fluid restriction (yes/no, amount),
- Health-related quality of life using the short form version of the Kansas City Cardiomyopathy Questionnaire (KCCQ-12) [22],
- Depressivity and anxiety using the ultrashort form of the German version of the Patient Health Questionnaire (PHQ-D) [12],
- Satisfaction with information transfer (NRS, 0 = not satisfied to 10 = very satisfied),
- Degree of informedness (NRS, 0 = not informed to 10 = very informed) and
- Adapted Patient Involvement in Care Scale (PICS) [18]

In addition, process-related data such as questions about thirst counselling, interventions implemented to alleviate thirst as well as sociodemographic and disease-related data will be assessed. Table 1 shows all outcome parameters according to the measurement time points.

**Table 1:** Measurement parameters: before–after study

	t0	t1	t2
Age	x	–	–
Gender	x	–	–
Education	x	–	–
Social support	x	–	–
Weight	x	x	x
Current medications	x	x	x
LVEF	x	–	–
Fluid restriction and amount	x	x	x
Co-morbidities	x	–	–
NYHA classification	x	x	x
Weather (temperature)	x	x	x
Documentation analysis for recording thirst and used interventions	x	x	x
Thirst intensity (NRS)	x	x	x
Thirst distress (TDS-HF)	x	x	x
Thirst frequency	x	x	x
Dry mouth (XI)	x	x	x
Health-related quality of life (KCCQ)	x	–	x
Depressivity and anxiety (4-item PHQ-D)	x	–	x
Conducted interventions	x	x	x
Informedness	x	x	x
Thirst counselling	–	–	x
Satisfaction with information transfer	–	–	x
Adapted Patient Involvement in Care Scale (PICS)	–	–	x

*KCCQ* Kansas City Cardiomyopathy Questionnaire, *LVEF* Left ventricular ejection fraction, *NRS* Numeric rating scale, *NYHA* New York Heart Association, *PHQ‑D* Patient Health Questionnaire–Deutsch, *PICS* Patient Involvement in Care Scale, *TDS-HF* Thirst Distress Scale for patients with heart failure, *XI* Xerostomia inventory

### Data collection

Baseline data will be collected before the training of nurses. Patients who meet all inclusion criteria within the first 3 days after admission to the ward and who are expected to stay at least 14 days (based on nurses’ judgement) will be included in the study.

If the patients have provided informed consent, all disease-related data from the hospitals’ patient documentation system (Table 1) will be collected and a questionnaire will be handed out to the patients, which is completed independently by the patient. Seven and 14 days after inclusion, participants will receive further questionnaire and the above-mentioned data are retrieves from the patient’s file.

If the planned sample size is reached, training of nursing staff on the counselling intervention will be performed and all participants will receive individual counselling by the nursing staff. Follow-up data will be collected after a 7-day individual intervention period of each participant.

#### Nurse-based counselling intervention (Table 2)

At least 50% of the nurses of the included wards at the two university hospitals who have a minimum workload of 19.25 h per week (50%) will be trained for the counselling intervention. Counselling will include the following content: basic information about thirst as a symptom of CHF and CHF therapy, assessment of thirst (occurrence, quality, intensity, distress), interventions already used by patients, suggestions for preventive interventions, shared decision making on interventions for individual participants. Patients will also receive information materials after counselling and nurses will record counselling in the patient chart.

**Table 2:** Intervention description based on the Template for Intervention Description and Replication (TIDieR) Checklist [9]

*BRIEF NAME*	Nurse-based counselling to promote self-care competencies concerning thirst in patients with advanced chronic heart failure eligible or listed for heart transplantation
*WHY*	The intervention aims to enable patients with advanced chronic heart failure eligible or listed for heart transplantation to choose and perform individual interventions to reduce thirst. The intervention is based on the systematic literature review, interviews with patients as well as the focus group and survey with nurses. The situation-specific theory of Heart Failure Self-Care is used as theoretical framework [16, 17]. Counselling follows a solution-focused approach [4, 27]
*WHAT*	*Contents of counselling:* assessment of thirst (occurrence, quality, intensity, distress); interventions already used by participants; thirst and heart failure; preventive interventions; shared decision making; use of information materials; documentation of the counselling
*WHO PROVIDES*	Trained staff nurses
*HOW*	Individual face-to-face counselling
*WHERE*	On the ward
*WHEN and HOW MUCH*	One counselling session within 7 days after inclusion
*TAILORING*	Personalized counselling, due to different individual perception of thirst and various possible interventions
*HOW WELL*	Patient evaluation after 7 and 14 days

### Economic outcomes

For cost analysis of the research project, cost components will be collected prospectively. Costs for materials and training, duration of intervention, time resources of the nursing staff, number of employees involved in the project and additional interventions that result from the counselling concept will be captured.

## Process evaluation

For the process evaluation, interviews will be conducted with patients who received counselling, a survey with nurses who performed counselling and with physicians involved in the provision of care on the wards. In addition, contextual factors will be assessed. Fidelity of the intervention will be assessed using a short protocol.

### Interviews with patients who received counselling

Five randomly selected patients per included hospital (*n* = 2) who received counselling by nurses will be asked to take part in an interview. The individual interviews will be conducted after t1as. The interviews will focus on patients’ experiences with the provision of information and counselling, the applicability of the recommended interventions to alleviate thirst, as well as possible influencing factors (promoting and inhibiting factors) of the implementation.

### Survey with nurses

Six months after the start of the implementation of the counselling concept, nurses who received the training will be invited to fill out a questionnaire, addressing the number of counselling sessions and their duration, the learning effect of the training, practicability of contents, framework conditions, influencing factors, facilitating factors and barriers as well as applicability of the concept.

### Survey with physicians

Also, 6 months after the start of the implementation of the counselling concept, treating physicians will receive an adapted questionnaire assessing knowledge, attitude, assessment of effects of the counselling concept.

### Context factors

To describe context factors on the wards included in the study, the following data will be collected:

- Nurses’ experience levels and qualifications,
- Number and characteristics of patients on the ward and
- Description of the environment in which counselling takes place

In addition, after the nurse training, protocols will be created in order to compare planning and actual implementation of the training.

### Data analysis

Quantitative data will be analysed using Statistical Package for Social Science (SPSS) Statistics (IBM Corp. Released 2022, IBM SPSS Statistics for Windows, Version 29.0.0.0, Armonk, NY, USA). The analysis will initially be performed descriptively and results will be presented as absolute and relative frequencies. Ordinal and metric data will be checked for normal distribution and shown as median (IQR) or mean (standard deviation).

The analysis of the measurement instruments TDS-HF, XI, KCCQ, PHQ‑D and PICS will be conducted both at item level and at scale level. In order to be able to detect differences due to the implementation of the counselling concept, depending on the data level, either a t-test, Mann–Whitney U test, chi-square (Χ^2^) test according to Pearson or exact test according to Fisher will be performed. Mean values of thirst intensity (NRS) of the individual measurement points will be evaluated as interrupted times series analysis using regression analyses.

Transcribed qualitative data (interviews with patients) will be analysed using content analyses [11] applying MAXQDA Standard 2022 (VERBI GmbH, Berlin, Germany). As recommended in the MRC Framework, quantitative and qualitative data will be combined related to the process relevant outcomes variables to evaluate factors that may have an influence on the implementation of the intervention [14].

## Ethical considerations and study approval

For study participation, written and informed consent is required. The ethics committee of the medical faculty of the Ruhr University Bochum, based in East Westphalia, has already approved the study (No. 2022-897). In addition, the study has been registered prospectively at German Clinical Trials Register (DRKS00029184 and DRKS00031440).

## Discussion

Development and implementation of an evidence-based counselling intervention to promote self-care competencies related to thirst in hospitalised patients with advanced CHF waiting for heart transplantation constitutes a complex intervention, as the intervention is dependent on different participants (e.g. nurses, patients and physicians), is implemented in a complex hospital context, and has different outcome parameters. Moreover, the development and implementation of the counselling intervention is influenced by various factors, for example competence of the nurses to perform a consultation, prior knowledge of the patients or environmental factors [19]. For the implementation of complex interventions, it is important to consider all factors throughout the process from development to evaluation [14]. Adjustments may be necessary during the study process. Process evaluation should be used to identify facilitating and hindering factors in the implementation process [14]. The main aim of implementing a counselling intervention is to improve self-care competencies and reduce patients’ symptom burden. There are currently only few intervention studies that have tested single interventions with the aim of thirst reduction in patients with CHF [3, 7, 10]. Complex interventions were not studied.

## Conclusion

The perception of thirst and strategies to reduce thirst are very different. Therefore, an individual approach is necessary. The current study aims to access preliminary data on the effects of a counselling intervention. The results can be used as a basis to develop and perform a subsequent randomized controlled trial.

### Supplementary Information

SPIRIT 2013 Checklist

## Abbreviations

CHF: Chronic heart failure
IQR: Interquartile range
KCCQ-12: Kansas City Cardiomyopathy Questionnaire-12
(L)-VAD: (Left) ventricular assist device
MRC: Medical research council
NRS: Numeric rating scale
PHQ‑D: Patient Health Questionnaire–Deutsch
PICS: Patient Involvement in Care Scale
SPSS: Statistical Package for Social Science
TDS-HF: Thirst Distress Scale for patients with heart failure
XI: Xerostomia inventory

## Declarations

For this study all investigations on humans described were carried out with the approval of the responsible ethics committee, in accordance with national law and the declaration of Helsinki in 1975 (in the current, revised version). This study is registered at German Clinical Trials Register as DRKS00029184 and DRKS00031440.

## References

[1] Allida SM, Hayward CS, Newton PJ (2018). Thirst in heart failure: what do we know so far?. Curr Opin Support Palliat Care.

[2] Allida SM, Inglis SC, Davidson PM (2016). A survey of views and opinions of health professionals managing thirst in chronic heart failure. Contemp Nurse.

[3] Allida SM, Shehab S, Inglis SC (2021). A RandomisEd ControLled TrIal of ChEwing Gum to RelieVE Thirst in Chronic Heart Failure (RELIEVE-CHF). Heart Lung Circ.

[4] Bowles N, Mackintosh C, Torn A (2001). Nurses’ communication skills: an evaluation of the impact of solution-focused communication training. J Adv Nurs.

[5] Bui QM, Allen LA, Lemond L (2019). Psychosocial evaluation of candidates for heart transplant and ventricular assist devices: beyond the current consensus. Circ Heart Fail.

[6] Chan AW, Tetzlaff JM, Gotzsche PC (2013). SPIRIT 2013 explanation and elaboration: guidance for protocols of clinical trials. BMJ.

[7] Chen Y, Ding J, Xi Y (2023). Thirst in heart failure: a scoping review. Nursing Open.

[8] Gong J, Waldreus N, Hu S (2022). Thirst and factors associated with thirst in hospitalized patients with heart failure in China. Heart Lung.

[9] Hoffmann TC, Glasziou PP, Boutron I (2014). Better reporting of interventions: template for intervention description and replication (TIDieR) checklist and guide. BMJ.

[10] Hudiyawati D, Suswardany DL (2021). Evaluating frozen strawberries as a strategy for thirst management in patients with congestive heart failure (CHF). IIUM Med J Malaysia.

[11] Kuckartz U (2014). Mixed Methods. Methodologie, Forschungsdesigns und Analyseverfahren.

[12] Löwe B, Wahl I, Rose M (2010). A 4-item measure of depression and anxiety: validation and standardization of the patient health questionnaire-4 (PHQ-4) in the general population. J Affect Disord.

[13] Mcdonagh TA, Metra M, Adamo M (2021). 2021 ESC guidelines for the diagnosis and treatment of acute and chronic heart failure. Eur Heart J.

[14] Moore GF, Audrey S, Barker M (2015). Process evaluation of complex interventions: medical research council guidance. BMJ.

[15] Reilly C, Meadows K, Dunbar S (2010). Thirst and QOL in persons with heart failure. Heart Lung.

[16] Riegel B, Dickson VV, Faulkner KM (2016). The situation-specific theory of heart failure self-care: revised and updated. J Cardiovasc Nurs.

[17] Riegel B, Jaarsma T, Lee CS (2019). Integrating symptoms into the middle-range theory of self-care of chronic illness. Ans Adv Nurs Sci.

[18] Scheibler F, Freise D, Pfaff H (2004). Die einbeziehung von patienten in die behandlung. J Public Health (Berl).

[19] Skivington K, Matthews L, Simpson SA (2021). A new framework for developing and evaluating complex interventions: update of medical research council guidance. BMJ.

[20] Spaderna H, Smits JM, Rahmel AO (2007). Psychosocial and behavioural factors in heart transplant candidates—an overview. Transpl Int.

[21] Spaderna H, Zahn D, Pretsch J (2013). Dietary habits are related to outcomes in patients with advanced heart failure awaiting heart transplantation. J Card Fail.

[22] Spertus JA, Jones PG (2015). Development and validation of a short version of the Kansas City cardiomyopathy questionnaire. Circ Cardiovasc Qual Outcomes.

[23] Thomson WM, van der Putten GJ, de Baat C (2011). Shortening the xerostomia inventory. Oral Surg Oral Med Oral Pathol Oral Radiol Endod.

[24] van der Wal MH, Jaarsma T, Moser DK (2010). Qualitative examination of compliance in heart failure patients in The Netherlands. Heart Lung.

[25] van der Wal MHL, Waldreus N, Jaarsma T (2020). Thirst in patients with heart failure in Sweden, the Netherlands, and Japan. J Cardiovasc Nurs.

[26] Waldréus N (2016). Thirst in patients with heart failure: description of thirst dimensions and associated factors with thirst.

[27] Webster DC (1990). Solution-focused approaches in psychiatric/mental health nursing. Perspect Psychiatr Care.

[28] Wefer F, Inkrot S, Waldréus N (2018). Thirst in German hospitalized patients with heart failure.

[29] Wefer F, Inkrot S, Waldreus N (2022). Translation and psychometric evaluation of the German version of the thirst distress scale for patients with heart failure. J Cardiovasc Nurs.

